# Increases in the soil ammonia oxidizing phylotypes and their rechange due to long-term irrigation with wastewater

**DOI:** 10.1371/journal.pone.0299518

**Published:** 2024-04-11

**Authors:** Eduardo J. Aguilar-Rangel, Alba Savin-Gámez, José Q. García-Maldonado, Blanca Prado, María Soledad Vásquez-Murrieta, Christina Siebe, Rocío J. Alcántara-Hernández

**Affiliations:** 1 Posgrado en Ciencias Biológicas, Universidad Nacional Autónoma de México, Unidad de Posgrado, Edificio D, 1° Piso, Circuito de Posgrados, Ciudad Universitaria, Coyoacán, 04510, Ciudad de México, México; 2 Departamento de Recursos del Mar, Centro de Investigación y de Estudios Avanzados del Instituto Politécnico Nacional, Unidad Merida 97310, Yucatán, México; 3 Instituto de Geología, Universidad Nacional Autónoma de México, Ciudad Universitaria, Av. Universidad 3000, Del. Coyoacán, 04510, Ciudad de México, México; 4 Escuela Nacional de Ciencias Biológicas, Instituto Politécnico Nacional, Del. Miguel Hidalgo, 11340, Ciudad de México, México; Fudan University, CHINA

## Abstract

Wastewater irrigation is a common practice for agricultural systems in arid and semiarid zones, which can help to overcome water scarcity and contribute with nutrient inputs. Ammonia-oxidizing bacteria (AOB) and archaea (AOA) are key in the transformation of NH_4_^+^-N in soil and can be affected by variations in soil pH, EC, N and C content, or accumulation of pollutants, derived from wastewater irrigation. The objective of this study was to determine the changes in the ammonia oxidizing communities in agricultural soils irrigated with wastewater for different periods of time (25, 50, and 100 years), and in rainfed soils (never irrigated). The *amoA* gene encoding for the catalytic subunit of the ammonia monooxygenase was used as molecular reporter; it was quantified by qPCR and sequenced by high throughput sequencing, and changes in the community composition were associated with the soil physicochemical characteristics. Soils irrigated with wastewater showed up to five times more the abundance of ammonia oxidizers (based on 16S rRNA gene relative abundance and *amoA* gene copies) than those under rainfed agriculture. While the *amoA*-AOA: *amoA*-AOB ratio decreased from 9.8 in rainfed soils to 1.6 in soils irrigated for 100 years, indicating a favoring environment for AOB rather than AOA. Further, the community structure of both AOA and AOB changed during wastewater irrigation compared to rainfed soils, mainly due to the abundance variation of certain phylotypes. Finally, the significant correlation between soil pH and the ammonia oxidizing community structure was confirmed, mainly for AOB; being the main environmental driver of the ammonia oxidizer community. Also, a calculated toxicity index based on metals concentrations showed a correlation with AOB communities, while the content of carbon and nitrogen was more associated with AOA communities. The results indicate that wastewater irrigation influence ammonia oxidizers communities, manly by the changes in the physicochemical environment.

## Introduction

Water scarcity has become a major concern of public policies around the globe. The 2030 Water Resources Group and the UN have forecasted shortfalls of nearly 40% of this resource by 2030 [1]. In arid and semiarid regions the problem is even worse, since less than 1000 m^3^ of available water per capita per year is projected for 2050 [2]. In order to face the water crisis in these regions, many institutions are proposing to reuse reclaimed water or wastewater for urban, industrial and especially agricultural purposes [3].

Irrigation of crop fields with partially treated wastewater can satisfy not only water requirements, but also those of nutrients, since depending on the treatment degree it also supplies organic matter and reduced nitrogen forms (NH_4_^+^-N and organic-N) to the plants [4]. Nevertheless, the prolonged use of wastewater fosters the accumulation of pollutants such as metal(oid)s or xenobiotics in the soils [5, 6], and can affect the soil physicochemical characteristics, depending on the water quality. For example, when untreated wastewater is used, soil organic carbon, total nitrogen, phosphorous, salinity, sodicity and metal(oid)s concentrations increase over time [7, 8]. These soil physicochemical disturbances also induce changes in the microbial community and biogeochemical profile of the irrigated soils. Agricultural rainfed soils have shown a similar bacterial community structure to those in the nearby shrubland; while the communities in those irrigated with wastewater differs, with increasing abundance of Proteobacteria and Bacteroidetes [9]. The 16S rRNA gene-based predictions suggest changes in N-cycle process because of the introduction of wastewater in these soils, such as increases in the potential of denitrification, confirmed with activity assays [5]. However, the changes in the functional community structure of microorganisms involved in the N-cycle deserve more detailed attention, due to the environmental implications of excess in N-application to crops and the consequent pollution of groundwater or air, with nitrate leaching [10] or by N_2_O emissions [11], respectively.

Reclaimed water or wastewater contains several N-forms (mainly NH_4_^+^ and organic N), and many soil prokaryotes associated to the N-cycle will be affected depending on the irrigation water quality. The microbial communities involved in the redox reactions of the N-cycle have shown to be sensible to pH changes, carbon and nitrogen sources and concentration, salinization or even contaminants. Ammonia oxidation is carried out by ammonia-oxidizing microorganisms (AOMs) and is a central step in the global nitrogen cycle. AOMs comprise Ammonia Oxidizing Bacteria (AOB), Ammonia Oxidizing Archaea (AOA), Complete Ammonia Oxidizers (comammox), and Anaerobic Ammonia Oxidizing Bacteria AnAOB [12]. AOA and AOB are primordial in nitrification and in the N-cycle, and these groups are widely distributed in different niches but sensible to physicochemical variations, so they are considered as soil quality markers [13–15].

The relationships between the ammonia oxidizers and the environment have been widely studied. pH is considered to be one of the main drivers for ammonia oxidizer community composition: in some soils the presence and abundances of ammonia oxidizing archaea (AOA) such as *Nitrososphaera* and *Nitrosotalea* are associated with low pH values (pH ~4–6) [16, 17], while those of *Nitrosospira* (an ammonia oxidizing bacteria (AOB)) are associated with alkaline pH values [18, 19]. Also, AOA can grow autotrophically, while AOB are also able to grow mixotrophically or heterotrophically, as observed for *Nitrososphaera* and *Nitrosotalea* [20, 21], allowing them to compete in systems enriched with small concentrations of organic compounds. For example, in some studies, soil organic carbon contents correlate negatively with the size of the AOA community, while the AOB community has a greater ecophysiological diversity and covers a broader range of habitats [22]. The ammonia oxidizers also have shown response to different fertilization regimes, as the resistance and resilience observed in AOA communities for organic amendments or in AOB communities after the application of inorganic N-fertilizers [23]. However, the dominance of *Nitrosospira* species such as *N*. *briensis*, who is known by its survival strategies in environments with fluctuating NH_4_^+^ environments, supports that some AOB can adapt to different niches [24]. In addition, the concentration of N-species is correlated to abundance and community structure of AOA and AOB. For example, at sites where ammonia is scarce (*e*.*g*., oceans), AOA such as *Nitrosopumilus maritimus* tend to be more abundant, while some AOB as *Nitrosomonas* tend to be adapted to large concentrations of ammonia [25]; on the other hand, a negative effect of NO_3_^-^ on AOA has been reported in soils [26]. Finally, pollutants also play a determinant role for ammonia oxidizer communities, where antibiotics can have a negative effect on AOB but not on AOA (36); and high zinc contents affected AOA while AOB showed a greater resilience [27].

In the current study, we evaluated the influence of prolonged wastewater irrigation on the ammonia-oxidizing microbial community of agricultural soils. The objective was to determine the changes in the community composition, abundance, and diversity of AOB and AOA in soils irrigated for 25, 50, and 100 years with wastewater irrigation, and to compare them with those of rainfed agricultural soils (0 years). The *amoA* gene from AOA and AOB was selected as a molecular marker for barcoding analysis, as this encodes for the catalytic domain of ammonia monooxygenase [28], and has been largely used as a genetic marker. Additionally, physicochemical soil properties were registered and associated with the changes in the ammonia oxidizer community. We hypothesize that wastewater irrigation will gradually change the physicochemical conditions of the soil and will impact the microbial community including ammonia oxidizers, boosting their abundance, and altering the native community structure.

## Materials and methods

### Site description

The studied area is located in the Mezquital Valley (MV), a semi-arid region in Central Mexico 60 km north of Mexico City. It is placed in the High Mexican Plateau at 1700–2100 m above sea level. The average annual temperature is around 18°C, with a delimited rainy season from June to September with an average annual rainfall between 400 and 600 mm. The native xerophytic vegetation was substituted by an intensively managed agricultural system, where the main crops rotate between maize (*Zea mays*) and fodder crops like lucerne (*Medicago sativa*). The land-use change is a result of an irrigation scheme with untreated municipal wastewater from the Mexico City Metropolitan Area for several years.

The wastewater for irrigation is distributed through large open but mainly lined canals, and from these derived to the fields by secondary mainly unlined channels. Therefore, the agricultural area contains units with different age of irrigation depending on the date that the wastewater distribution net was constructed, generating an irrigation chronosequence scheme within the valley since this management started, more than a century ago [29]. Yet, some areas within the agricultural district are rainfed fields or freshwater irrigated crops using local wells [9]. Phaeozems are the dominant soils in the area, with the presence of Leptosols and Vertisols. These soils are in general neutral to slightly alkaline, where the wastewater irrigation process have favored the increases of soil organic matter stocks [30].

Some of the wastewater physicochemical characteristics and pollutants has been reported through time, yet this composition varies depending on the sampling date (S1 Table in S1 File). The pH values reported are from 6.7 to 8.9, an electrical conductivity (EC) of 930–2264 μS/cm, total organic carbon of 35–188 mg/L, and organic-N and NH_4_^+^ as main N forms, the presence of some metals and pharmaceuticals have been also determined. Wastewater irrigation is performed by field overflow, where each field receives a mean of 10 irrigations per year, each one of about 200 mm.

### Sampling and processing

To study the progressive effect of wastewater irrigation over ammonia oxidizers in agricultural soils, a chronosequence based on the irrigation time was established. Plots under this wastewater practice for 25, 50, and 100 years were selected, sampling three plots per irrigation interval, considering also three rainfed fields (0 years) as controls. The sampled soils were classified as Phaeozems, without crop cover at the sampling time, and collected in the Tula-03 irrigation district within a range of 16 km (S2 Table in S1 File). The rainfed site is managed under a traditional scheme with farmyard manure fertilization without information on the amount applied, where maize is cultivated once a year; the plots are at 2 km distance from the nearest fields recently irrigated with wastewater. The 25- and 50-years irrigated plots are at ~16 km distance from the control site, while the 100-years site is at 12 km from the rainfed site, and at 10 km from the 25- and 50-years irrigated plots. To ensure the representativeness of the site, the sampling design consisted of four equidistant longitudinal transects which were parallel to the irrigation channel, as it has been observed that pollutant concentration varies in parallel by the overflow irrigation process. Superficial bulk soil samples (0–10 cm) were collected with a corer (2.6 cm diameter) along each transect at 10 m distance, consisting of 25–33 cores per transect depending on the plot length. The soil cores collected at the four transects were mixed per plot, to obtain a composite sample per field (February 2020). The collected soil (0–10 cm) was deposited in sterile recipients and kept at 4°C in darkness for transportation to the laboratory (5 h). The soils were then sieved with a 2 mm mesh to remove rocks and vegetal material, and homogenized to keep representative fresh soil samples, which were collected in 50-mL sterile conical tubes and preserved at -20°C until DNA extraction. The remanent soil was processed for physicochemical characterization as mentioned below.

### Physicochemical characterization

The samples were air dried in the darkness for 72 h and subsequently homogenized and again sieved with a metallic mesh (2 mm) and stored. pH, and electrical conductivity (EC) were determined in air dried samples following standard protocols based on Sparks *et al*. [31]. Soil pH and EC were measured in saturation extracts.

For elemental characterization soil samples were grounded to pass through a 0.180 mm sieve. Total C (TC) and N (TN) were determined with a 2400 Series II CHNS Elemental Analyzer (PerkinElmer) calibrated with the LECO CNS 2000 standard. Organic C (OC) was determined in sample aliquots after removal of carbonates using 5 N HCl. Metals such as Cd, Co, Cu, Ni, Pb, and Zn were measured in HNO_3_-digested soil samples (Anton Paar Multiwave 3000 for 10 min) by ICP-OES (Perkin Elmer ICP-OES Optima 8300) [32].

### DNA extraction and PCR

Total DNA was extracted from 0.25 g of previously processed soil samples using PowerSoil® DNA isolation Kit (Qiagen) according to manufacturer instructions. The obtained DNA was then quantified using a Qubit 4 Fluorometer (ThermoFisher Scientific) with a Qubit dsDNA HS Assay Kit (ThermoFisher Scientific) and stored at -20°C for further analysis. Firstly, we employed a 16S rRNA gene amplicon sequencing approach to know the composition of the prokaryotic assemblages (Bacteria and Archaea) using the V4 region of the 16S rRNA gene with the primers 515F/806R (with a barcoded reverse primer, a MiSeq adaptors), following the protocols of Caporaso et al. [33], the PCR conditions and amplification protocols can be consulted in Aguilar-Rangel *et al*. [34].

The *amoA* genes from AOA and AOB were amplified with the primers Arch-amoAF/Arch-amoAR [35] for AOA, and amoA-1F/ amoA-2R [36] for AOB. The primers contain additional specific MiSeq-tailed sequences. PCR reactions were 25 μL containing: template DNA (~15 ng per reaction), 1× PCR buffer (Mg^2+^ free), 1.5 mmol/L MgCl_2_, 0.4 μM of each primer, 200 μM of each deoxynucleotide triphosphate, dimethyl sulfoxide 5%, and 1 U of ExTaq DNA polymerase (Takara ExTaq) (Takara Bio Inc.). The amplification protocols consisted in i) an initial step of denaturation at 95°C (5 min), followed by ii) 35 cycles of amplification including a denaturing step (95°C, 1 min), an annealing step (specific for each primer set, 1 min) and an extending step (72°C, 1 min), and iii) a final extension at 72°C for 10 min. The annealing temperature of *amoA* of AOA was 53°C and 60°C for *amoA*-AOB. Dual indices were added to the amoA-amplicons with the Nextera XT Index Kit in a second PCR for eight cycles. Individual barcoded amplicons were diluted and purified in 10 mM Tris (pH 8.5) and pooled at 9 pM equimolar concentration.

Finally, the 16S rRNA amplicons were paired-end sequenced on an Illumina MiSeq platform at the Yale Center for Genome Analysis (CT, USA), while the *amoA* genes were single-end sequenced in a MiSeq platform with a MiSeq Reagent Kit V3 (2x250 cycles) at CINVESTAV-Mérida, Mexico.

### Sequences analysis

The analysis of the 16S rRNA and *amoA* gene sequences was performed with the QIIME2 (2021.4) pipeline (https://qiime2.org) [37], using the parameters reported in Aguilar-Rangel *et al*. [34]. The sequences were classified with the Silva database as reference (silva-138-99-515-806-nb-classifier.qza, released in November 2020) (http://www.arb-silva.de).

The *amoA* raw sequences were demultiplexed using the “q2-demux” plugin and then their quality was verified (314,755 raw reads obtained). Sequence filtering was carried out by the DADA2 method (trimming at 250 pb) resulting in 300 representative sequences or ASVs (sequences with 100% nucleotide identity) obtained from 144,696 filtered, denoised and non-chimeric reads [38]. Then, these ASVs were collapsed using the “q2-vsearch” plugin to generate OTU at 95% of identity according to Yu *et al*. [39]. A manual data curation was performed to confirm that every OTU was associated with previously reported *amoA* genes and no pseudogenes with stop codons were present. The curated data were rarefacted using the “rarefy_even_depth” tool from package ‘phyloseq’ (in the R platform) to the minimum number of total counts per sample resulting in 1,446 counts per sample for AOA and 2,587 for AOB, to standardize the same number of counts (S1 Fig in S1 File). The *amoA* sequences were aligned using MUSCLE method from SEAVIEW software [40]. From the multiple alignments, a Neighbor-Joining tree supported by Jukes-Cantor distance method was built for alpha and beta-diversity analysis, and for taxonomic affiliation, a new Neighbor-Joining using the K2P distance method, and 1,000 replicates were built.

BioSample metadata is available in the NCBI BioSample database (http://www.ncbi.nlm.nih.gov/biosample/) under accession numbers SAMN28921204, SAMN28921205, SAMN28921206 and SAMN28921207 (BioProject PRJNA847174). Raw data sequence data is available in SRA-NCBI database (https://www.ncbi.nlm.nih.gov/sra), with the accession numbers in the Supporting information material.

### Gene quantification

The *amoA* genes from AOA and AOB were quantified using SYBR green technology using total DNA from each sample as template. The assays were performed employing a StepOne Real-time PCR System (Thermo Fisher Scientific). A standard curve was built using serial dilutions of purified PCR products from extracted total DNA for each assay. Each reaction has a final volume of 10 μl and was prepared using 1× Premix Ex Taq Bulk (Takara Bio Inc.), 1× ROX Reference Dye, 0.2μmol/L of the forward and reverse primers and 1ng of template. The cycling program consists in one initial denaturing cycle at 95°C (5min); and 40 amplification cycles [95°C for 1min; an annealing temperature depending on the primer pair for 45 s, and 72°C for 30s]. At the end of the program a melt curve was generated to verify the quality of the products and the assay (absence of primer dimers and amplification of only one band). The program for the melt curve is: an initial step was 95°C for 15s, followed by a cycle of crescent temperature (+0.5°C every 15s), starting at 60°C and finalizing at 95°C for every amplicon.

### Statistical analysis

Diversity indexes calculation and principal coordinate analysis (PCoA) were performed using ‘phyloseq’ package from R (version 4.1.0). The PCoA analysis was supported by the weighted unifrac distance method. PERMANOVA test (999 permutations, paired) was implemented to found significant differences in community structure and their abundance using “betadisper” and “permutest” functions from the R package “vegan”. Spearman and Mann-Whitney probes were realized in GraphPad Prism 6 to identify statistical significance for environmental and biological parameters.

## Results

### Soil physicochemical and microbiological characteristics

The soils at the sampled sites have been previously classified as vertic or haplic Phaeozems [41]. Duration of wastewater irrigation influenced the physicochemical characteristics of the top soil (Table 1), where most of the measured properties increased with irrigation time including pH, electrical conductivity (EC), total carbon content (TC), total nitrogen (TN) and organic carbon (OC). The pH did not show a significant change with time, but the average value increased from 7.70±0.06 in the rainfed soils, to ranges between 8.4–8.5 in the irrigated soils. EC, and concentrations of TC, TN and OC increase between sites irrigated for 25 years and 50 years reaching almost twice the concentrations observed in rainfed fields, while concentrations of TC, TN and OC in soils irrigated for 100 years do not differ from those measured in soils irrigated for 50 years.

**Table 1:** Physicochemical characteristics of the sampled top soils.

Years	pH	EC (dS/m)	TC (%)	OC (%)	TN (%)	TI*
**0**	7.70 ± 0.06^a^	0.68 ± 0.09ª	1.22 ± 0.09^a^	1.17 ± 0.07^a^	0.13 ± 0.01^a^	375 ± 41^a^
**25**	8.42 ± 0.04^b^	1.95 ± 0.23^b^	1.99 ± 0.41^b^	1.82 ± 0.44^ab^	0.20 ± 0.04^ab^	587 ± 120^a^
**50**	8.48 ± 0.03^b^	2.36 ± 0.88^b^	2.45 ± 0.27^b^	2.18 ± 0.31^b^	0.23 ± 0.02^b^	764 ± 119^a^
**100**	8.51 ± 0.11^b^	1.42 ± 0.08^ab^	2.26 ± 0.14^b^	2.06 ± 0.22^b^	0.24 ± 0.02^b^	2164 ± 312^b^

Different letters indicate significant differences among fields with different duration of irrigation (*p* < 0.05)

* TI (Toxicity index) was calculated based on Azarbad *et al*. [42]

The calculated toxicity index (TI) based on the equation proposed by Azarbad *et al*. [42], which is based on the metal concentration in the soil shows a progressive increase with duration of irrigation years, resulting in an almost 7-fold larger value in soils irrigated for 100 years in comparison with rain-fed soils. Mainly, the concentration of Cu, Ni, Pb and Zn increased through years peaking at 100 years (S3 Table in S1 File).

### AOA and AOB abundance

**Fig 1:**
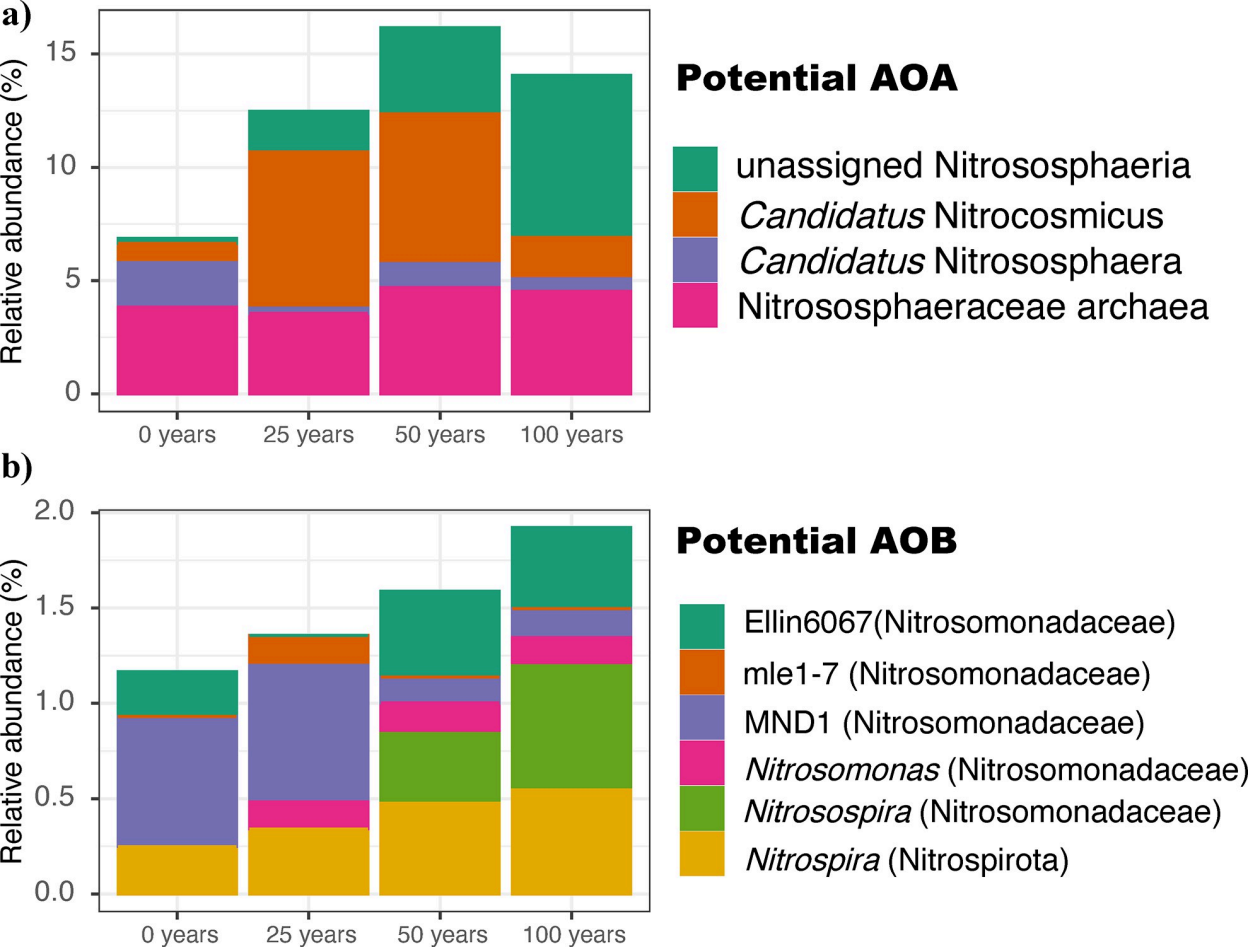
Relative abundance of potential ammonia oxidizers by a 16S rRNA gene survey. a) Potential AOA. b) Potential AOB.

Duration of wastewater irrigation affected the relative abundance of the prokaryotic communities associated with ammonia oxidation processes, here assessed by 16S rRNA and *amoA* genes. Firstly, the 16S rRNA gene survey showed that the relative abundance of AOA (Nitrososphaeria) increased from 6.86% in the rainfed soils, to 12–16% in the irrigated soils; while the potential AOB slightly increased from 1.16% in the rainfed soils, to 1.92% in the soils irrigated for 100 years (Fig 1).

**Fig 2:**
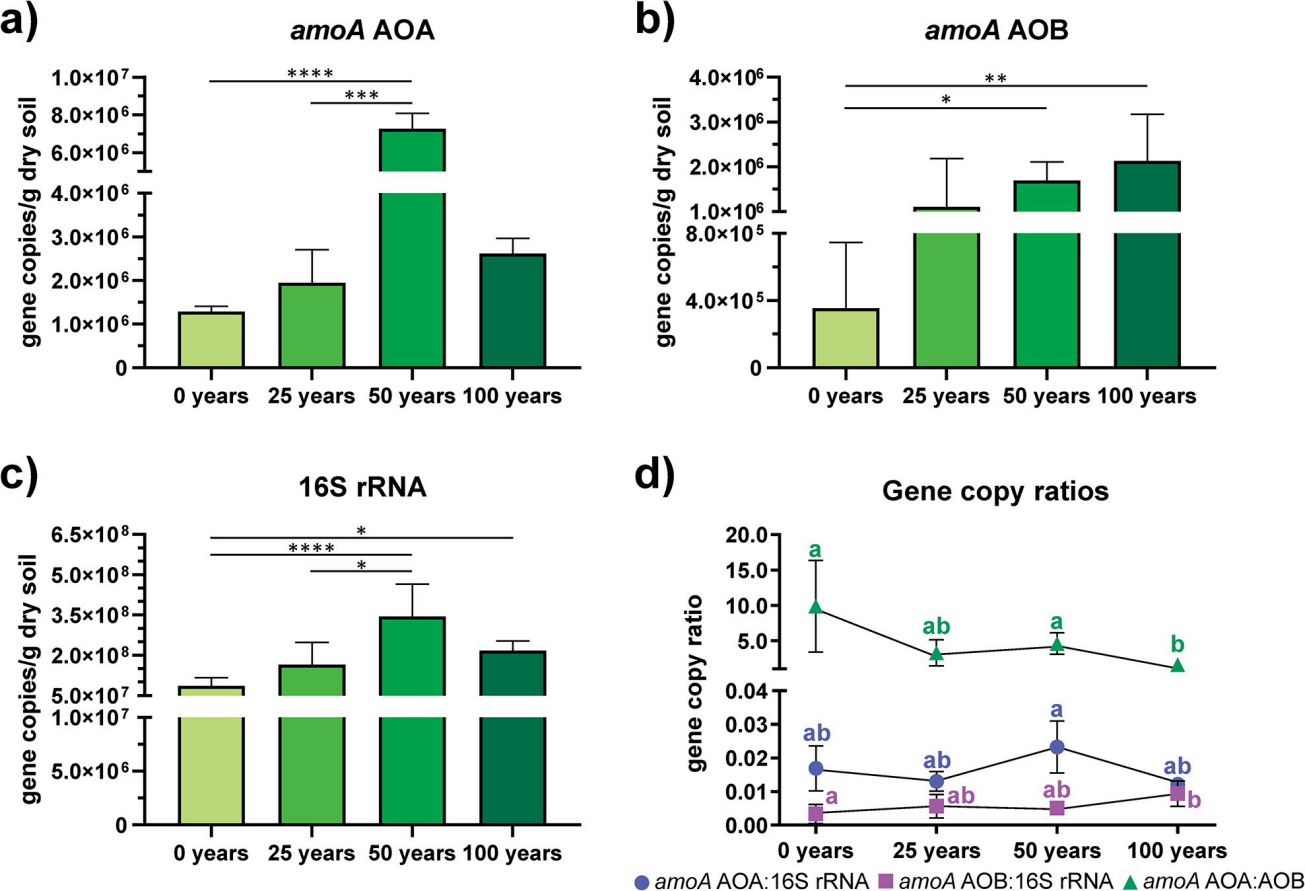
Quantification of *amoA* and 16S rRNA genes in the soil samples at different irrigation times. a) *amoA*-AOA gene. b) *amoA*-AOB gene. c) 16S rRNA gene. d) Gene copy ratios.

The quantification of *amoA* genes confirmed this trend, where the number of *amoA-*AOA gene copies was highest in the soils irrigated for 50 years (7.27 × 10^6^ *amoA* copies / g dry soil), followed by the soils irrigated for 25 and 100 years (Fig 2A). The *amoA-*AOB gene copies ranged from 1.11 × 10^6^–2.13 × 10^6^ *amoA* copies / g dry soil in the soils irrigated with wastewater, increasing significantly after 25 years under irrigation, and keeping a similar number of gene copies thereafter (Fig 2B). These gene counts indicate that AOA are more abundant than AOB in all the studied soil, but especially for the wastewater irrigated soils. The number of 16S rRNA gene copies also increased significantly in the irrigated samples, by almost one order of magnitude (Fig 2C). Therefore, to know the real increment in relation with the microbial biomass, ratios between *amoA* and 16S rRNA gene abundance were calculated. The number of archaeal *amoA* gene copies related to the 16S rRNA gene copies (*amoA* AOA:16S rRNA) do not vary through time, except for 50 years where the ratio was significantly larger (Fig 2D). While the changes in the *amoA* AOB:16S RNA ratio suggest a constant increase until 50 years but increasing significantly after 100 years. Further, the relation between AOA and AOB *amoA* genes showed a reduction in the relation to almost 1.6:1 copies of AOA over AOB during 100 years of irrigation (Fig 2D).

### Ammonia-oxidizing community diversity and structure

**Fig 3:**
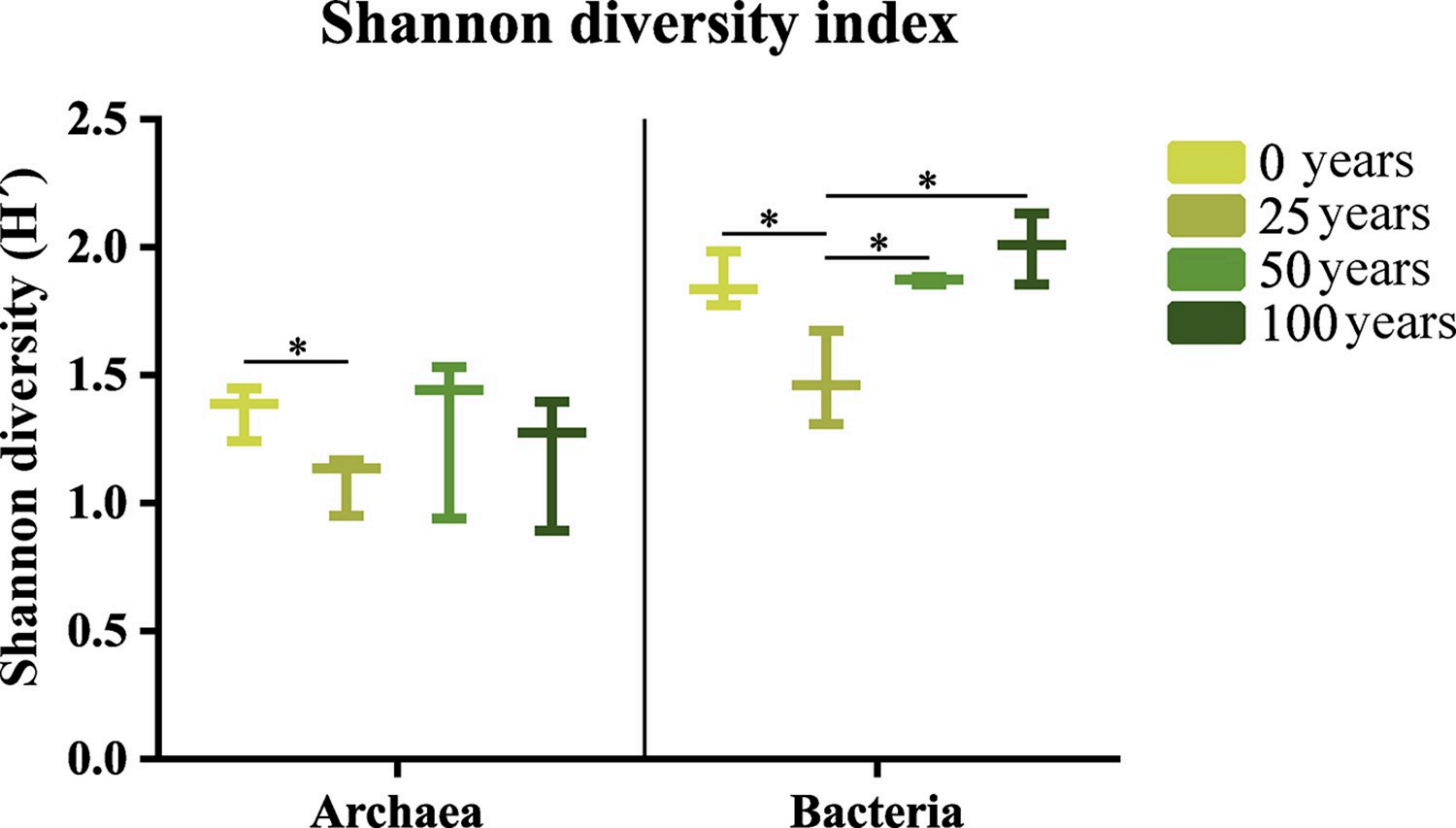
Shannon Diversity index (*H*’) based on *amoA*-AOA (left) and *amoA*-AOB sequences (right).

The composition of the archaeal and bacterial ammonia-oxidizing communities was analyzed using the sequences of the *amoA* genes by Illumina MiSeq. The *amoA-*AOA gene sequences showed a higher number of ASVs (154 ASVs) than the *amoA*-AOB (112 ASVs). Nevertheless, the *amoA-*AOB sequences showed a higher diversity than its AOA counterpart, based on the Shannon index (Fig 3). For both groups, the detected diversity is significantly smaller (*p*-value < 0.05) in the soils irrigated for 25 years in comparison to the rainfed fields. Only for AOB, a gradual increase in the diversity of *amoA-*AOB genes can be noticed as duration of irrigation increases from 25 to 100 years (Fig 3).

**Fig 4:**
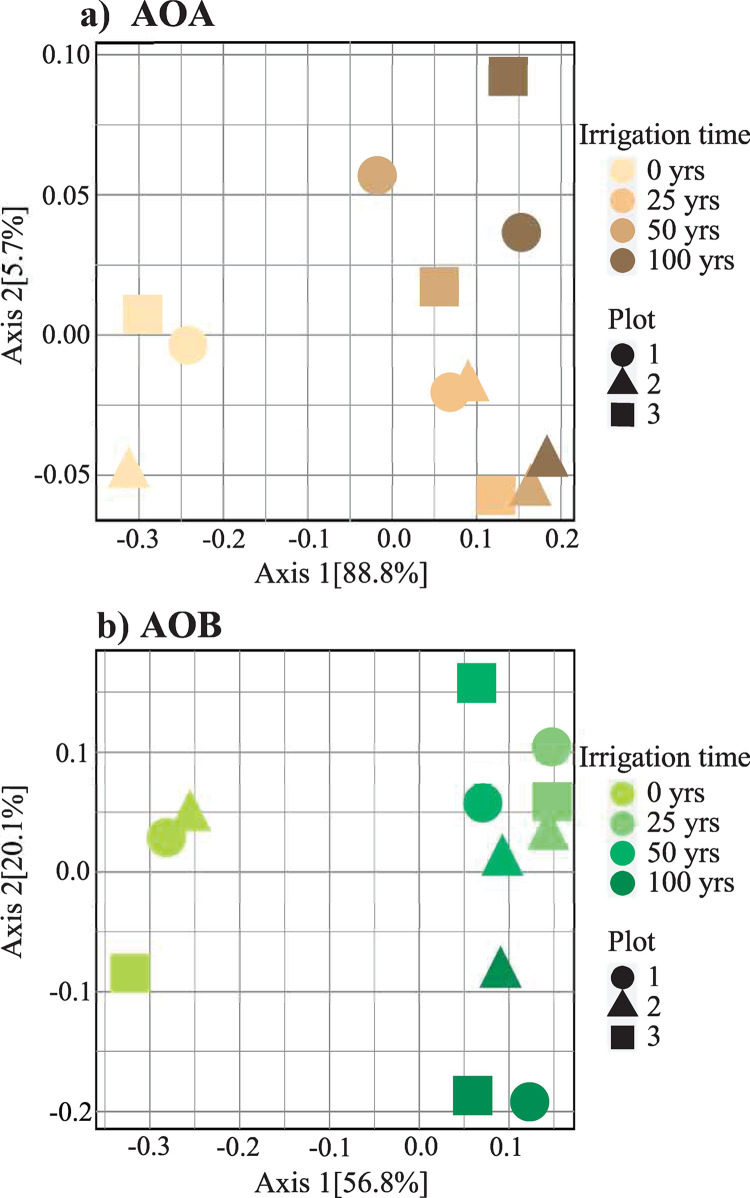
Community structure of ammonia oxidizers in soils irrigated with wastewater for 25, 50 and 100 years, and a rainfed irrigated soil (0 years). Weighted PCoA-UniFrac of a) AOA, and b) AOB, based on *amoA* sequences.

The ammonia-oxidizing community structure also changed according to the duration of irrigation with wastewater based on the presence and abundance of the sequences (Fig 4). In the case of *amoA*-AOA communities, the use of wastewater for irrigation changed the community structure, separating the samples from rainfed fields from the irrigated samples in the ordination analysis (Fig 4A). A similar situation was found for the *amoA*-AOB communities (Fig 4B), where the rainfed and wastewater irrigated soils were separated between them, but not among the duration of irrigation (i.e., 25, 50 and 100 years), indicating that the structure of the community of ammonia oxidizers is similar in mid to long term irrigated soils. In general, it was observed that AOA communities from rainfed soils are significantly different from wastewater irrigated soils, and AOB are tending to be different but in a lesser degree (S4 Table in S1 File).

### Taxonomic affiliation of the *amoA* gene sequences

**Fig 5:**
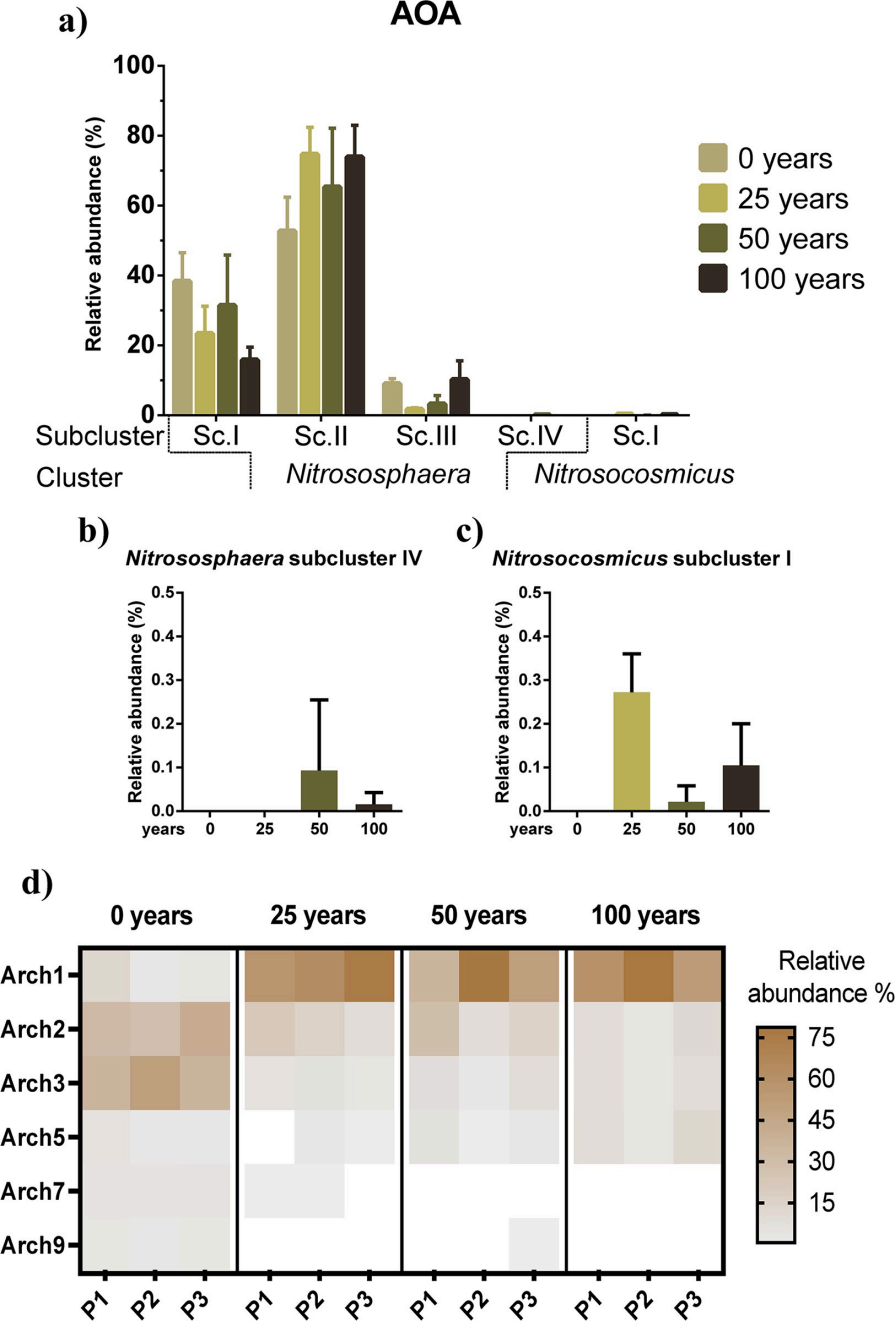
Relative abundance of archaeal *amoA* clusters and the detected OTUs based on their phylogenetical associations. a) Archaeal ammonia oxidizers groups; b) *Nitrososphaera* subcluster IV; c) *Nitrosocosmicus* subcluster I; d) heatmap from AOA representative OTUs. The identity of the OTUs is defined by the prefix Arch (*v*.*g*., Arch1).

The *amoA* gene sequences were collapsed in OTUs with 95% identity, resulting in 22 OTUs for AOA. Then, a phylogenetic tree was constructed to affiliate the sequences with other ones reported in databases. The archaeal *amoA* sequences were clustered within two groups related to the *Nitrososphaera* and *Nitrosocosmicus* genera (S2 Fig in S1 File). The *Nitrososphaera* group contained most of the sequences, representing >99% of the relative abundance of archaeal *amoA* genes found. This group was further divided into four subclusters, of which three were related to uncultured samples (subclusters I, II and IV), and one to *Nitrososphaera viennensis*, *Ca*. Nitrososphaera everglandensis and *Nitrososphaera gargensis* (subcluster III) (Fig 5A). The *Nitrososphaera* subclusters I, II, and III contained most of the phylotypes at all the irrigation conditions, and with no significant differences among them (Fig 5A). In contrast, the *Nitrososphaera* subcluster IV contained just one phylotype (<0.1% of relative abundance) (Fig 5B), which was found only in soils irrigated for 50 and 100 years with wastewater. Similarly, the *Nitrosocosmicus* subcluster I appeared in the irrigated soils (Fig 5C), at a low relative abundance (<0.3%) but was absent in the rainfed soils. Although no significant changes on these major archaeal groups belonging to *Nitrososphaera* were detected, it was possible to observe variations in their OTUs (S3 Fig in S1 File). For example, Arch1 and Arch5 seem to increase with the duration of wastewater irrigation, while Arch2 and Arch3 diminish, or are below the detection limits, as Arch7 and Arch9 (Fig 5D).

**Fig 6:**
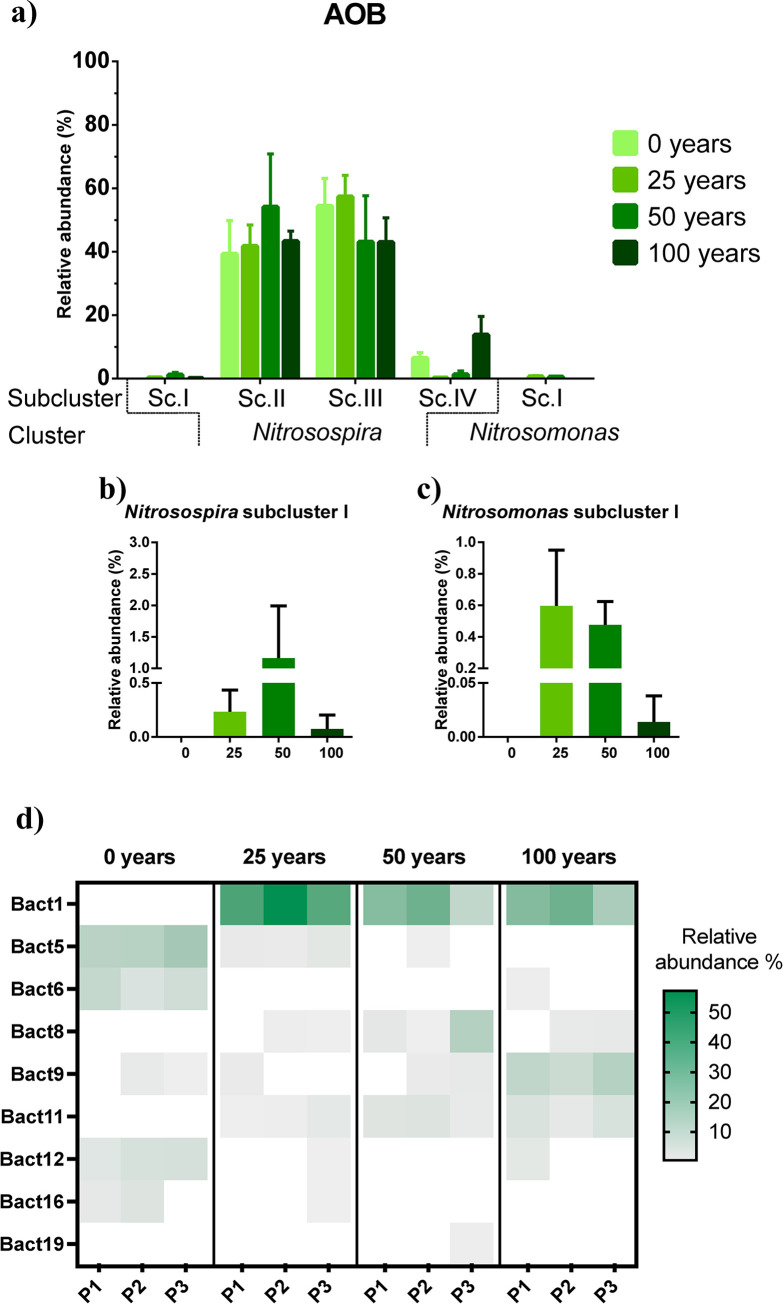
Relative abundances of bacterial *amoA* clusters and the detected OTUs based on their phylogenetical associations. a) Bacterial ammonia oxidizers groups; b) *Nitrosospira* subcluster I; c) *Nitrosomonas* subcluster I; d) heatmap from AOB representative OTUs. The identity of the OTUs is defined by the prefix Bact (*v*.*g*., Bact1).

The 30 OTUs of ammonia oxidizing bacteria were separated in two groups associated with *Nitrosospira* and *Nitrosomonas*, being the first one the most abundant with 29 phylotypes and representing >99% of the relative abundance (S4 Fig in S1 File). This *Nitrosospira* group was divided in four subclusters named according to the association with: *Nitrosospira* sp. NpAV and *Nitrosospira lacus* (subcluster I), *Nitrosovibrio* sp. Nv4 and *Nitrosospira multiformis* (subcluster II), environmental clones of *Nitrosospira* (subcluster III), and *Nitrosovibrio tenuis* and *Nitrosospira briensis* (subcluster IV). The *Nitrosospira* subclusters II, III and IV were found in all the irrigated fields representing most of the *amoA*-AOB sequences, with a relative abundance of 45, 49, and 5.4% in average, respectively; and did not differ significantly among fields irrigated for different length of time (Fig 6A). Though soils irrigated for 50 years show largest abundances in subcluster II, and decreasing abundances as irrigation length increases in subcluster III. The *Nitrosospira* subcluster I and the *Nitrosomonas* subcluster I only contained OTUs from the soils irrigated with wastewater, and with a maximum relative abundance of 2 and 1%, respectively (Fig 6B and 6C). The *Nitrosomonas* cluster only contained one OTU, closely related to *Nitrosomonas mobilis* and *Nitrosomonas stercoris*, and represented <0.6% of relative abundance in average (Fig 6C, S4 Fig in S1 File). Similar to Archaea, the bacterial *amoA*-OTUs also showed a differentiation by phylotype according to the duration of irrigation (S5 Fig in S1 File). The *Nitrosospira-*like OTUs such as, Bact1 and Bact9 presented a large increase in the wastewater irrigated soils with respect to rainfed soils, up to 185 and 19-times, respectively (Fig 6D), while other ones such as Bact8, Bact11 and Bact19, were only detected in irrigated soil. Bact5, Bact6, Bact12 and Bact16, tended to decrease as the soils are irrigated with wastewater or were not detected (Fig 6D).

### Association of AOA and AOB with the soil physicochemical properties

**Fig 7:**
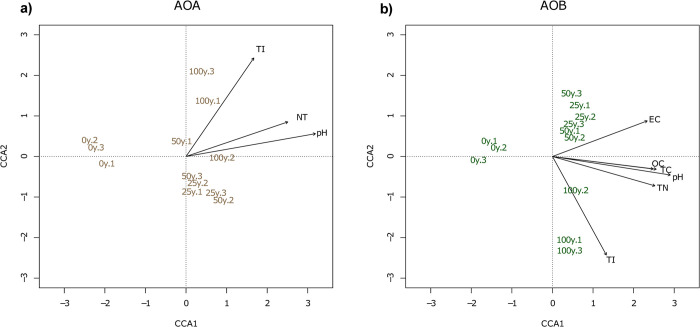
Canonical Correlation Analyses (CCA) of the ammonia oxidizing communities and their significant correlations with the soil physicochemical characteristics. a) for AOA; and b) for AOB. The nomenclature of all samples corresponds to irrigated years and the number of the sampled plot (*v*.*g*., 0y.1).

Canonical correspondence analysis (CCA) was carried out to assess relations between the AOA and AOB community structures and their physicochemical soil environment, based on the *amoA* sequences. The *amoA*-AOA communities formed two clusters, one containing the communities found in rainfed soils, and one with the communities found in wastewater irrigated soils. The irrigated soils were significantly associated with properties such as pH, TN and the toxicity index (TI), the latter was particularly related with the soils irrigated for 100 years (Fig 7A). The *amoA*-AOB community presented three clusters consisting of: i) rainfed soils, ii) soils irrigated for 25 and 50 years, and iii) soils irrigated for 100 years (Fig 7B). The physicochemical properties explaining the observed grouping were those of the wastewater irrigated soils, similar to the observations found for the AOA community, where the soils irrigated for 100 years was closely correlated to the TI.

**Fig 8:**
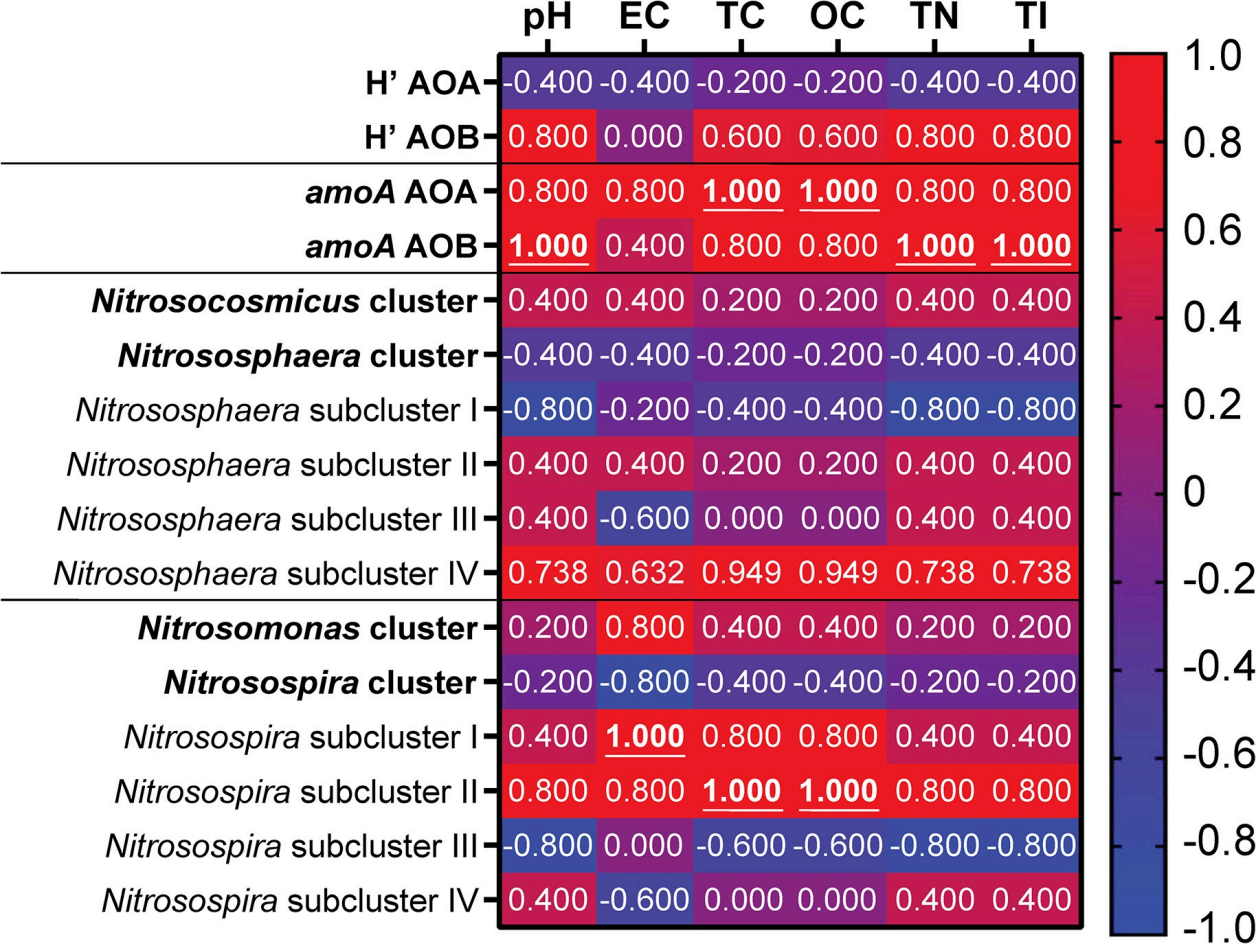
Heatmap with the Spearman correlation among physicochemical soil properties and ammonia oxidizing groups in the studied soils. Underlined numbers represent those with *p*-values < 0.05.

In addition, a Spearman correlation test was performed to analyze the correlation between the abundance and diversity of AOA and AOB, with the soil physicochemical characteristics (Fig 8). For diversity, there was no significant correlation between the Shannon index and the surveyed soil properties, although a correlation of 0.8 was found between the AOB *H’* and pH, TN and TI. The abundance of *amoA* gene copies did show positive significant correlations, where AOA was correlated with carbon forms (TC and OC), while AOB correlated with pH, TN and TI. In relation to the AO taxonomic affiliation, there was not a significant correlation between the different AOA groups with the environmental attributes; however, *Nitrososphaera* subcluster I stands out for a negative correlation between pH, TN and TI with a value of 0.8. In the case of AOB, *Nitrosospira*-subcluster I had a significant positive correlation with EC, besides a strong correlation with carbon sources (0.8), while the *Nitrosospira*-subcluster II correlated with carbon (TC and OC), and showed a strong positive correlation with pH and CE (of 0.8). Finally, *Nitrosospira*-subcluster III despite not showing significant correlations, there were negative robust ones with pH, TN and TI (Fig 8). Finally, at OTU level, there was a strong correlation with some of the physicochemical properties, without a clear tendency. For AOA-OTUs, two phylotypes belonging to different *Nitrososphaera* subclusters presented a significative correlation (p values <0.05) with the concentration of soil carbon (TC and OC), one of them positive (Arch4), and another one negative (Arch.7). AOB phylotypes presented a similar behavior, since some OTUs significantly correlate positively with pH, TN and TI (Bact10 and Bact11), while one presented a negative correlation (Bact5) with these same soil characteristics (S5 Table in S1 File).

## Discussion

Most of the microbiological studies on wastewater reuse in agriculture have monitored the innocuity and environmental risks of the cultivated crops [29], but only few studies have assessed the impacts on the soil microbial community [43, 44], and their potential biogeochemical profile. In the studied area, Friedel *et al*. [5] determined microbial biomass and respiration as well as some enzyme activities in topsoil samples irrigated for different length of time. While Broszat *et al*. [45] identify that wastewater irrigation increases the potential pathogens after the irrigation for 100 years, and Lüneberg *et al*. [9] showed the changes in the structure and functionality of the bacterial. In this study, we considered to survey a microbiological key functional soil group, such as the ammonia oxidizers after continuous irrigation for 100 years with organic N compounds and ammonia contained in wastewater, cycling the excess of N compounds in the agrosystem. These communities showed changes in their number, composition, and taxonomic affiliation, which can be related to long term changes in some of the soil’s properties, as here showed.

Municipal wastewater from Mexico City has been increasingly used for irrigation of agricultural fields over the last century. Previous studies based on functional predictions suggest that processes such as nitrification can be increased due to irrigation with wastewater, in comparison to the use of freshwater from local wells [9]. This trend was also found with the 16S rRNA gene survey here done where the relative abundance of potential ammonia oxidizers increased with irrigation time in the long term (Fig 1), and then confirmed with the increase of *amoA* absolute counts with length of irrigation (Fig 2). The gene abundance of *amoA* is generally used as estimator of the amount of ammonia oxidizers in a system, and therefore their variations can be indicators of environmental changes. AOA abundance doubles in soils irrigated for 100 years, from 1.28 × 10^6^ to 2.61 × 10^6^ *amoA* copies per gram of dry soil, showing similar values to those found in grassland soils contaminated with metals for 80 years, or even paddy soils exposed to wastewater irrigation [46, 47]. However, it is notable that the *amoA*-AOA abundance peaks in soils irrigated for 50 years, and it is correlated with the increase in carbon forms (TC and OC). This assumption is attributed to the mixotroph lifestyle of some AOA, as some have the capability to assimilate organic carbon [48, 49].

Ibekwe *et al*. [44] reported that the use of treated wastewater did not influence the microbial diversity in comparison with freshwater irrigated soils in an agricultural experiment station, but the 16S rRNA gene sequences related to N-cycling groups such as nitrifying, nitrogen-fixing and denitrifying bacteria, increased their abundance when treated wastewater was used instead of freshwater. In this study, a similar result was observed using *amoA* genes, where we did not observe significant changes on AOA and AOB diversity through time determined by the Shannon diversity index (Fig 3); but the relative and absolute abundance of ammonia oxidizers increased in soils irrigated for long time with wastewater compared to the rainfed soils. Further, by sequencing these *amoA* genes, we detect a change on AOA and AOB community structure, where the samples of the rainfed sites were different from those irrigated with wastewater, and with no significant differences according to duration of irrigation (i.e., 25, 50 and 100 years) (Fig 4).

To date, there is not enough information about the direct influence of the use of raw wastewater to irrigate agricultural soils over the ammonia oxidizing community, nonetheless, fertilization experiments could be considered an analogous approach since wastewater is similar to an organic fertilization [8]. Previous reports have suggested that AOA and AOB respond differently to external stimuli such as N-fertilization [48, 50]. For example, the community structure of AOA has been reported as generally unaffected by fertilizer addition in forest soils in the long-term [51], yet Chen *et al*. [52] showed changes in the community changes in acid paddy soils. Our results showed that AOA community structure was different in the rainfed soils compared to the wastewater irrigated ones; suggesting that soil characteristics and management could be key to understand how archaeal ammonia oxidizers adapt to soil environmental changes.

AOB community structure is presumably more sensitive to fertilizer input in the short-term and long-term [51, 53]. Wertz *et al*. [51] found that periodic fertilization over forest acid soils increased potential AOB, with a significant correlation with nitrate concentration. While Wu *et al*. [53] results indicate that fertilization during 22 years on paddy soils modified AOB community structure in relation to non-fertilized plots, besides the bacterial *amoA* gene counts increases almost 50 times after the constant fertilization with urea and organic matter (rice straw). In this study, gradual changes in the AOB community structure were detected but in a gradual scale, where the AOB community of the rainfed soil was different from that in the wastewater irrigated ones, and among them, the irrigated for 25–50 years cluster in a different group than irrigated for 100 years. Furthermore, an increase of almost one order of magnitude were detected in the *amoA* gene copies from AOB on soils irrigated by 100 years with wastewater in relation with rainfed soils.

On behalf of the *amoA* AOA sequences, we distinguished the two most common archaeal groups from soil, *Nitrososphaera* and *Nitrosocosmicus* [54]. *Ca*. Nitrososphaera spp. was the most dominant cluster in the studied soils as was expected, since it is recognized as the more abundant AOA in soils, particularly in those with agricultural managements [55, 56]. Previous studies in arable soils have demonstrated changes in subcluster composition of a *Nitrososphaera* cluster, depending on variations on climatical conditions such as temperature or precipitation [57]. However, Habteselassie *et al*. [58] have not found differences between archaeal *amoA* sequence clustering in soil under different fertilization regimes. In concordance with this study, the current survey also did not find significant differences in the abundance of the *Nitrososphaera* subclusters according to duration of irrigation; however, it was possible to detect the following tendencies. *Nitrososphaera* subcluster I, that grouped uncultured representatives from superficial agricultural soils, showed a diminution in their relative abundance after 100 years irrigation compared to the rainfed soil, which could be attributed to the diminution of the prevalent phylotype Arch2, associated to an uncultured archaeon from agricultural soil. While *Nitrososphaera* subcluster II, which is associated with uncultured strains from paddy and restored crop soils, was the most abundant one in all the sampled soils, mainly due to the presence of the phylotype Arch1. This *Nitrososphaera* subcluster increased slightly in wastewater irrigated soils, though the conforming OTUs showed diverse behaviors, the most abundant phylotype (Arch1) augmented up to 9 times in the irrigated soils, while Arch3 had a lower abundance in irrigated soils than in rainfed ones. Phylotype Arch3 was closely related to a clone from a restored farm soil of a semiarid region [59], therefore, its presence and abundance might be negatively affected by intensive agricultural management. The *Nitrososphaera* subcluster III abundance was surprisingly low as expected, since it contains *amoA* sequences that belong to the widespread *Nitrososphaera* subcluster 1.1 [60]. The other widespread genus from terrestrial AOA is *Ca*. Nitrosocosmicus [61] was also detected, nevertheless their affiliated sequences only were found in soils irrigated with wastewater, which is consistent with their proximity with *Nitrosocosmicus franklandus*, an ureolitic and high-ammonia tolerant archaea whose ideal growth conditions are at pH values of 7.5 [62].

Bacterial *amoA* sequences are associated with *Nitrosospira*, which has been reported as the most dominant bacterial ammonia oxidizer genus in soil, and *Nitrosomonas* which is less common [58]. The *Nitrosomonas* cluster is only present after wastewater irrigation; this genus is a common representative of agricultural soils irrigated with wastewater effluents [63], yet only one OTU is associated (Bact15). This OTU is closely related to *Nitrosomonas stercoris*, an AOB that tolerates high concentrations of ammonium, isolated from composted cattle manure, and found in wastewater and wastewater treatment plants [64]; thus, OTU Bact15 could be introduced by the wastewater used for irrigation. Finally, it is worth to notice that the 16S rRNA gene survey suggests a larger proportion of potential AOB belonging to *Nitrosomonas*, therefore there could be a methodological bias by primer coverage [65], so further studies must continue to verify the relevance of *Nitrosomonas* in soils under wastewater irrigation.

The *Nitrosospira* cluster was dominant in the studied soils and was divided into four subclusters, where the abundance of these subclusters they did not change significantly during the different irrigation lengths, comparably to the trend observed in the AOA *Ca*. Nitrososphaera cluster. Subcluster I was only detected on wastewater irrigated soils and it was closely related to a previously established cluster, which includes *Nitrosospira* species from water-saturated or moisture-rich soils [66], being consistent with the irrigation techniques used on the Mezquital Valley. The mean abundance of subcluster II increased during irrigation years, which can be related to bacteria such as *Nitrosospira multiformis* found in fertilized agricultural soils [67], or *Nitrosovibrio* sp. Nv4 whose main habitat is wastewater [68] and possibly adapted to the constant irrigation by water of this quality. In this subcluster, Bact2 and Bact4 are dominant as was expected by its proper ubiquitous distribution on agricultural soils according to its phylogenetical relation with *N*. *multiformis* and isolates from agricultural soils derived from alluvial deposits respectively [69, 70]. The subcluster III was the other dominant group, containing different uncultured *Nitrosospira* and which showed a diminution of nearly 10% in the mean abundance at 50 and 100 years. Regardless that this subcluster has a similar abundance in rainfed soil and in soil irrigated for 25 years, their OTU content varies, since in rainfed soil it is dominated by Bact5 and then is substituted by Bact1 after 25 years of irrigation, a close phylotype related to an isolate from riparian soils. This phylotype replacement is continuously found through the years in the wastewater irrigated soils, possibly due to environmental adaptations as Bact1 its closely related to an isolate from a riparian zone, which is known to be enriched with nutrients and pollutants [71]. Furthermore, other abundant phylotypes were exclusively in the rainfed soils such as Bact6 and Bact16, which were affiliated to sequences from restored agricultural soils and river isolates, respectively, both systems with a similar nitrogen input and a circumneutral pH [59, 72]. Finally, the *Nitrosospira* subcluster IV was present in all studied irrigation samples as expected by their association with uncultured soil strains with positive effects of N fertilization, and *Nitrosovibrio tenuis* Nv1 [73].

As we observed, pH is a primary driver in the determination of the niche of the ammonia oxidizers. Previous studies in agricultural soils irrigated with wastewater have measured a short-term drop in pH between 0.2–0.7 units at short-term (after 3 hours of wastewater irrigation) [10], as well as in the long-term between 0.3–3.5 units [74, 75]. Nonetheless, our results registered a gradual increase of pH as duration of irrigation proceeds and particularly in comparison with rainfed soils, as other author have described previously, particularly in agricultural soils [76] or in soils close to unprotected wastewater ponds and enriched with N-compounds by deposition [77]. This pH increase could be attributed to a high Na_2_CaCO_3_ concentration of soils due to salinization caused by the input of salts by the wastewater salts input [78]. A similar effect could be observed in EC possibly due to the salts introduced by wastewater, whose main ions have shown to be Na^+^, Cl^-^ and HCO_3_^-^ [34]. The peak of EC in soil saturation extracts was after 50 years of irrigation with 2.36 dS/m, considered as very slight saline and therefore, it can influence the microbiota in a minor way [79]. The increase in EC by wastewater irrigation has been also found in agricultural soils treated with olive mill wastewater [80], by the constant input of different ions for two years. In the Mezquital Valley soils, this increase in EC has been also found due to the water quality used for irrigation [9], and in Mollic Vertisols and Eutric Leptosols irrigated with untreated wastewater for different lengths of time (between 0 and 80 years) [5]. In this study, the soils from fields irrigated for 50 and 100 years showed an increased content of OC, TC, and TN up to 86%, as observed on agricultural soils irrigated with reclaimed wastewater [81]. The here observed gradual increase of soil TN tended to a saturation asymptote, while TC and OC peaked after 50 years under irrigation possibly due to the dynamic equilibrium between mineralization and humification, that results from an increase in primary productivity [30]. Additionally, previous studies in the zone found that rainfed agricultural soil have smallest OC stock in comparison with soil irrigated for 40 years with wastewater irrigated soils (which are 1.5 times higher), as well as the quality of the organic matter changing from hydrophobic to hydrophilic compounds such as cellulose and lignin (with modified structure) [30]. TI was calculated from the concentration of total metals in the soil and indicates the accumulation of pollutants with duration of irrigation. The index increased with irrigation length, since municipal wastewater used for irrigation contains trace concentrations of these potentially toxic elements [6]. Previous studies in the zone have confirmed that continuous irrigation leads to the accumulation of metals such as Hg, Cr, As or Pb, especially in the superficial soil layer [82].

Long term wastewater irrigation has changed soil quality in Mezquital Valley affecting several characteristics that include, variations in soil pH, the increase of CE and C and N stocks, and the input of pollutants. All these changes influence the structure community of ammonia oxidizers, particularly by the specific niches of these microorganisms [49, 83]. The use of wastewater for irrigation was determinant in the establishment of AOA and AOB, due to the N-inputs, and the settled community of both groups are well adapted the current physicochemical environment. Nevertheless, our results suggest that AOB tend to be favored by the conditions generated by the use of wastewater in comparison with AOA.

The changes in the ammonia oxidizing communities seem to be linked to the changes in the soil characteristics depending on the ammonia oxidizers groups and phylotypes. For example, as was expected AOA abundance significantly correlates with TC and OC probably by their suggested mixotroph lifestyle [49], considering that prior studies have found that AOA could uptake organic carbon sources coming from the wastewater [48]. Nevertheless, despite the advantages that confer mixotrophy to AOA, their abundance could be limited by the raising metal pollution, the increment of pH or the disadvantaged competence with AOB, due to the high input of ammonia from wastewater irrigation [25, 84]. The number gene copies of *amoA-*AOB in rainfed soils here studied was barely lower than those in not impacted soils [85], but this number gradually increased up to 10-fold in the long term irrigated soils reaching a number similar to agricultural soils after irrigation with reclaimed wastewater [86]. It should be emphasized that the AOB abundance in the community is significantly higher in long term irrigated crop fields than in the rainfed soil, probably by the accumulative input of reduced N-forms and soil pH values which confer selective advantages to the AOB [18]. This finding is supported by the AOA:AOB ratio, though AOA is more abundant in these kind of soils–and as expected–[83], the AOA:AOB ratio decreases progressively as the duration of irrigation with wastewater goes on. The diminution of the ratio has been previously described in soils exposed to industrial waste effluents [87], and it was accompanied by an increase in pH, TC, TN, OC and the accumulation of pollutants of different nature, such as metals and aromatic hydrocarbons, as here found. In conformity with our results, the AOB abundance correlates positively with pH, TN and TI, which is calculated using the concentration of metals in the soil. Particularly, it was observed the effect of metals over ammonia oxidizing communities. High Zn concentrations in alkaline crop soils have significant effect over AOA and AOB abundance, while Cu at 50 mg/kg concentration does not have significant effect over AOB but AOA [88]. Other studies have confirmed a positive correlation between Cu, Co, Ni, Cd, Pb and Zn over *Nitrospira* and *Nitrosomonas*, and negative over AOA and *Nitrosospira*, this last is contrary to our results were *Nitrosospira* is dominant [89]. Yet, these results also suggested that the response to environmental changes is different for each AOA or AOB phylotype, indicating that the functional responses to environmental alterations for our studied sites are phylotype-specific.

## Conclusions

Wastewater irrigation represents a good strategy for zones with water scarcity, yet it is relevant to know the effects of this practice in the long term, considering N-cycling microbial groups that are related to soil biogeochemistry. In this study, the ammonia oxidizing communities showed changes correlated with the physicochemical characteristic of the soils in response to wastewater irrigation, such as carbon concentration, pH, EC and the content of accumulable pollutants (TI). After 100 years of irrigation with wastewater, it was observed that archaeal and bacterial ammonia oxidizers abundance and community structure changed. Nevertheless, despite the ammonia oxidizer core persist through time, new phylotypes were detected which seemed to be better adapted to the changing conditions of the soil; the abundance of AOB over AOA can be favored by the environmental conditions produced by wastewater irrigation until reaching an equilibrium. This suggest that these soil systems should be monitored further, especially when new management practices are considered such as changing the water quality of the irrigation by treating it in wastewater treatment plants that use chloride to diminish the presence of potential pathogens, besides considering other soil types within the agricultural district. These new investigations must be carried out to clarify the effect over other N-related groups such as nitrite oxidizing bacteria or denitrifiers.

## Supporting information

S1 File Tables (S1 to S5 Tables) and figures (S1 to S5 Figs). (DOCX)

S2 File The accession numbers for the raw sequence database. (XLSX)

S1 Graphical abstract (TIF)
