# Supplementary material for: Increases in the soil ammonia oxidizing phylotypes and their rechange due to long-term irrigation with wastewater
Source: PLoS One. 2024 Apr 11;19(4):e0299518. doi: 10.1371/journal.pone.0299518 (PMC11008854; doi:10.1371/journal.pone.0299518)
Supplement: S1 File — (DOCX) [file pone.0299518.s001.docx]

**Increases in the soil ammonia oxidizing phylotypes and their rechange due to long-term irrigation with wastewater.**

Eduardo J Aguilar-Rangel^1^, Alba Savin-Gámez^1^, José Q. García-Maldonado^2^, Blanca L Prado^3^, María Soledad Vásquez-Murrieta^4^, Christina Siebe^3^, Rocío J Alcántara-Hernández^3*^

^1^Posgrado en Ciencias Biológicas, Universidad Nacional Autónoma de México, Unidad de Posgrado, Edificio D, 1° Piso, Circuito de Posgrados, Ciudad Universitaria, Coyoacán, 04510, Ciudad de México, México.

^2^Departamento de Recursos del Mar, Centro de Investigación y de Estudios Avanzados del IPN, Unidad Merida 97310, Yucatán, México.

^3^Instituto de Geología, Universidad Nacional Autónoma de México, Ciudad Universitaria, Av. Universidad 3000, Del. Coyoacán, 04510, Ciudad de México, México.

^4^Escuela Nacional de Ciencias Biológicas, Instituto Politécnico Nacional, Del. Miguel Hidalgo, 11340, Ciudad de México, México.

Running head: Ammonia oxidizing microorganisms in agricultural soil

*Corresponding author: Rocío J Alcántara-Hernández. Instituto de Geología, Universidad Nacional Autónoma de México, Ciudad Universitaria, Av. Universidad 3000, Del. Coyoacán, 04510, Ciudad de México, México, E mail: [ralcantarah@geologia.unam.mx](mailto:ralcantarah@geologia.unam.mx), [rocio.alcantara.h@gmail.com](mailto:rocio.alcantara.h@gmail.com) Telephone: +52 (55) 56224286 Ext. 154. Fax: +52 (55) 5550 6644.

**S1 Table Wastewater physicochemical characteristics and pollutants composition in the Mezquital Valley reported through time.**

| **Year** | **pH** | **EC** | **N-forms** | **C-forms** | **Pharmaceuticals** | **Metals and metalloids** | **Reference** |
| --- | --- | --- | --- | --- | --- | --- | --- |
| 1985 | 8.4 | 1483 μS/cm | TN: 29.3 mg/L  NH_4_^+^- N: 17.5 mg/L  NO_3_^-^- N: 0.015 mg/L |  |  | Pb: 0.11 mg/L  Cd: 0.005 mg/L  Cu: 0.15 mg/L  Zn: 0.40 mg/L  Cr: 0.11 mg/L | Siebe [1] |
| 1991 |  |  |  |  |  | Cu: 0.117 mg/L  Mn: 0.323 mg/L  Zn: 0.018 mg/L  Cd: 0.005 mg/L  Cr: 0.112 mg/L  Co: 0.044 mg/L  Ni: 0.086 mg/L  Pb: 0.068 mg/L | Cajuste *et al.* [2] |
| 1996 |  |  |  |  |  | Cd: 0.05 mg/L  Ni: 0.95 mg/L  Pb: 0.13 mg/L | Vázquez-Alarcón *et al.* [3] |
| 1997-1998 |  | 1,437–1,6891 μS/cm | TN: 37-38 mg/L  NH_4_^+^- N: 24–32 mg/L  NO_3_^-^- N: 0 – 1 mg/L | TOC: 35–188 mg/L |  | As: 0 - 0.008 mg/L  Cu: 0.05 - 0.07 mg/L  Cr: 0 - 0.04 mg/L  Fe: 1 - 1.2 mg/L  Mn: 0.03 - 0.2 mg/L  Pb: 0.09–0.1 mg/L | Jimenez and Chávez [4] |
| 2004 |  |  |  |  | Trimethoprim: 0.28 – 0.31 μg/L  Clarithromycin: 0.40 – 0.80 μg/L  Clindamycin: 0.07 – 0.12 μg/L  Erythromycin: 0.03 – 0.07 μg/L  Metoprolol:2.4 – 3.1 μg/L  Ibuprofen: 0.42 – 0.54 μg/L  Naproxen: 4.80 – 5.60 μg/L  Diclofenac: 0.4 – 0.55 μg/L  Sulfasalazine: 0.4 – 0.438 μg/L  Bezafibrate: 0.08 – 0.1 μg/L  Gemfibrozil: 0.02 – 0.22 μg/L |  | Siemens *et al.* [5] |
| **S1 Table (*Continuation*)** | | | | | | | |
| **Year** | **pH** | **EC** | **N-forms** | **C-forms** | **Pharmaceuticals** | **Metals and metalloids** | **Reference** |
|  |  |  |  |  |  |  |  |
| 2007 |  |  |  |  |  | Fe: 0.13 – 2.30 mg/L  Cu: 0.012 – 0.12 mg/L  As: 0 – 0.76 mg/L  Mn: 0.14 – 0.15 mg/L  Pb: 0.021 – 0.051 mg/L  Zn: 0.024 – 0.46 mg/L | Lesser-Carrillo *et al.* [6] |
| 2011 | 7.5-8 |  |  | DOC: 15 – 80 mg/L |  | Fe: 100 – 250 μg/L  THg: 0.01 – 0.04 μg/L  MMHg: 0.002 – 0.02 μg/L  Cr: 0.5 – 4 μg/L  Pb: 100 – 300 μg/L  As: 5 – 20 μg/L | Guédron *et al.* [7] |
| 2012-2014 | 7.78 – 8.02 |  | TN: 44 – 53 mg/L  NH_4_^+^- N: 26 – 31 mg/L  NO_3_^-^- N: 0 – 0.01 mg/L | DOC: 47 – 50 mg/L |  |  | Hernández-Martínez *et al.* [8] |
| 2013 |  |  |  |  | Acetaminophen: 13.9 – 49.2 μg/L  Carbamazepine: 0.25 – 0.35 μg/L  Ciprofloxacin: 0.81- 2.57 μg/L  Erythromycin: 0.94 – 1.08 μg/L  Lincomycin: 2.13 – 2.92 μg/L  Sulfamethoxazole: 4.53 – 6.57 μg/L  Gemfibrozil: 9.48 – 23.6 μg/L  Ibuprofen: 0.84 – 1.07 μg/L  Naproxen: 6.12 – 18.6 μg/L  Metformin: 39.0 – 89.7 μg/L |  | Lesser *et al.* [9] |
| 2017-2018 | 6.7-8.9 | 930 – 2264 µS/cm | NH_4_^+^- N: 19.4 – 33.5 mg/L  NO_3_^-^- N: 0 – 3.3 mg/L | DOC: 180 mg/L  DIC: 6.4 mg/L |  |  | Aguilar-Rangel *et al.* [10] |

**References**

1. Siebe C. Heavy metal availability to plants in soils irrigated with wastewater from Mexico City. Water Science and Technology. 1995; 32(12): 29-34. <https://doi.org/10.1016/0273-1223(96)00135-7>

2. Cajuste LJ, Carrillo RG, Cota EG, Laird RJ. The distribution of metals from wastewater in the Mexican Valley of Mezquital. Water, Air, and Soil Pollution. 1991; 57(1): 763-71. <https://doi.org/10.1007/BF00282940>

3. Vázquez-Alarcón A, Justin-Cajuste L, Siebe-Grabach CD, Alcántar-González G, de la Isla de Bauer MdL. Cadmio, níquel y plomo en agua residual, suelo y cultivos en el Valle del Mezquital, Hidalgo, México. Agrociencia. 2001; 35(3): 267-74.

4. Jimenez B, Chávez A. Quality assessment of an aquifer recharged with wastewater for its potential use as drinking source: “El Mezquital Valley” case. Water Science and Technology. 2004; 50(2): 269-76. <https://doi.org/10.2166/wst.2004.0141>

5. Siemens J, Huschek G, Siebe C, Kaupenjohann M. Concentrations and mobility of human pharmaceuticals in the world's largest wastewater irrigation system, Mexico City–Mezquital Valley. Water Research. 2008; 42(8-9): 2124-34. <https://doi.org/10.1016/j.watres.2007.11.019>

6. Lesser-Carrillo LE, Lesser-Illades JM, Arellano-Islas S, González-Posadas D. Water balance and groundwater quality in the Valle del Mezquital aquifer, central Mexico. Rev mex cienc geol. 2011; 28(3): 323-36.

7. Guédron S, Duwig C, Prado BL, Point D, Flores MG, Siebe C. (Methyl)mercury, arsenic, and lead contamination of the world’s largest wastewater irrigation system: the Mezquital Valley (Hidalgo State—Mexico). Water, Air, & Soil Pollution. 2014; 225(8): 2045-. <https://doi.org/10.1007/s11270-014-2045-3>

8. Hernández-Martínez JL, Prado B, Cayetano-Salazar M, Bischoff W-A, Siebe C. Ammonium-nitrate dynamics in the critical zone during single irrigation events with untreated sewage effluents. Journal of Soils and Sediments. 2018; 18(2): 467-80. <https://doi.org/10.1007/s11368-016-1506-2>

9. Lesser LE, Mora A, Moreau C, Mahlknecht J, Hernández-Antonio A, Ramírez AI, Barrios-Piña H. Survey of 218 organic contaminants in groundwater derived from the world's largest untreated wastewater irrigation system: Mezquital Valley, Mexico. Chemosphere. 2018; 198: 510-21. <https://doi.org/10.1016/j.chemosphere.2018.01.154>

10. Aguilar-Rangel EJ, Prado BL, Vásquez-Murrieta MS, los Santos PE-d, Siebe C, Falcón LI, et al. Temporal analysis of the microbial communities in a nitrate-contaminated aquifer and the co-occurrence of anammox, n-damo and nitrous-oxide reducing bacteria. Journal of Contaminant Hydrology. 2020; 234: 103657. <https://doi.org/10.1016/j.jconhyd.2020.103657>

**S2 Table Localization of sampled sites**

| **Irrigation time** | **Plot** | **Coordinates** |
| --- | --- | --- |
| **0 years**  **(rainfed)** | 1 | 20º09’48.45”N; 99º06’25.76”W |
|  | 2 | 20º09’42.74”N; 99º06’25.29”W |
|  | 3 | 20º09’37.49”N; 99º06’24.99”W |
| **25 years** | 1 | 20º02’24.63”N; 99º11’38.52”W |
|  | 2 | 20º02’24.05”N; 99º11’35.15”W |
|  | 3 | 20º02’23.47”N; 99º11’33.40”W |
| **50 years** | 1 | 20º02’21.49”N; 99º11’56.38”W |
|  | 2 | 20º02’18.10”N; 99º11’59.29”W |
|  | 3 | 20º02’23.14”N; 99º11’54.40”W |
| **100 years** | 1 | 20º07’29.08”N; 99º12’47.80”W |
|  | 2 | 20º07’30.01”N; 99º12’51.23”W |
|  | 3 | 20º07’33.68”N; 99º12’48.60”W |

**S1 Fig.** **Rarefaction curves of the sequenced *amoA* genes.** a) AOA-*amoA* gene. b) AOB-*amoA* gene

**S3 Table Metal concentrations** **in rainfed soils (0 years) and soils irrigated with wastewater in the long term (25, 50 and 100 years).**

| **Irrigation** | **Plot** | **Cd**  (mg/kg) | **Co**  (mg/kg) | **Cu**  (mg/kg) | **Ni**  (mg/kg) | **Pb**  (mg/kg) | **Zn**  (mg/kg) |
| --- | --- | --- | --- | --- | --- | --- | --- |
| 0 years | 1 | 1.09 | 8.91 | 3.97 | 10.67 | 6.10 | 28.81 |
|  | 2 | 1.02 | 9.44 | 3.51 | 9.72 | 6.04 | 27.87 |
|  | 3 | 1.06 | 9.03 | 0.00 | 9.72 | 6.34 | 28.28 |
| 25 years | 1 | 1.12 | 8.16 | 6.52 | 13.21 | 7.25 | 44.84 |
|  | 2 | 1.30 | 8.54 | 10.20 | 14.88 | 7.80 | 53.78 |
|  | 3 | 1.20 | 9.05 | 4.23 | 14.23 | 6.63 | 36.70 |
| 50 years | 1 | 1.20 | 7.04 | 11.79 | 13.49 | 9.50 | 64.74 |
|  | 2 | 1.24 | 7.13 | 11.58 | 13.42 | 8.82 | 65.19 |
|  | 3 | 1.29 | 7.03 | 7.67 | 12.92 | 7.13 | 50.73 |
| 100 years | 1 | 1.99 | 7.72 | 36.05 | 23.10 | 35.46 | 152.38 |
|  | 2 | 2.08 | 7.86 | 32.32 | 21.39 | 31.11 | 138.14 |
|  | 3 | 1.71 | 8.18 | 27.18 | 18.97 | 26.69 | 111.19 |

**S4 Table *p*-values observed among the sampled sites considering the beta diversity analyses** (999 permutations). RF =rainfed soils

| **AOA unifrac** | | |  | **AOB unifrac** | | |
| --- | --- | --- | --- | --- | --- | --- |
|  | Wastewater irrigated soil | |  |  | Wastewater irrigated soil | |
| RF | **0.035** | Permuted |  | RF | 0.080* | Permuted |
|  | **0.020** | Observed |  |  | 0.075* | Observed |
| Pr(>F)= 0.018 | | |  | Pr(>F)= 0.074 | | |
| **AOA wunifrac** | | |  | **AOB wunifrac** | | |
|  | Wastewater irrigated soil | |  |  | Wastewater irrigated soil | |
| RF | 0.288 | Permuted |  | RF | 0.113 | Permuted |
|  | 0.260 | Observed |  |  | 0.089 | Observed |
| Pr(>F)= 0.310 | | |  | Pr(>F)= 0.071 | | |
| Bold numbers = *p* values <0.05; ^*^significant at *p*-value < 0.10 | | | | | | |

**S2 Fig** **Bio-Neighbor joining phylogenetic tree of *amoA* sequences from AOA obtained from collected chronosequence soil samples.** The tree was obtained with K2P distance method. Bootstrap values ≥50% (1000 replicates).

**S3 Fig** **Heatmap of the relative abundance of AOA OTUs in the plots that corresponds to the different chronosequence times.**

**S4 Fig** **Bio-Neighbor joining phylogenetic tree of amoA sequences from AOB obtained from collected chronosequence soil samples.** The tree was obtained with K2P distance method. Bootstrap values ≥50% (1000 replicates).

**S5 Fig** **Heatmap of the relative abundance of AOB OTUs in the plots that corresponds to the different chronosequence times.**

| **S5 Table Spearman correlation among physicochemical and OTUs from ammonia oxidizers** | | | | | | |
| --- | --- | --- | --- | --- | --- | --- |
|  | **pH** | **EC** | **TC** | **OC** | **TN** | **TI** |
| **AOA** |  |  |  |  |  |  |
| Arch1 | 0.400 | 0.400 | 0.200 | 0.200 | 0.400 | 0.400 |
| Arch2 | -0.800 | -0.200 | -0.400 | -0.400 | -0.800 | -0.800 |
| Arch3 | -0.200 | -0.800 | -0.400 | -0.400 | -0.200 | -0.200 |
| Arch4 | 0.800 | 0.800 | **1.000** | **1.000** | 0.800 | 0.800 |
| Arch5 | 0.800 | 0.000 | 0.600 | 0.600 | 0.800 | 0.800 |
| Arch6 | -0.800 | -0.200 | -0.400 | -0.400 | -0.800 | -0.800 |
| Arch7 | -0.800 | -0.800 | **-1.000** | **-1.000** | -0.800 | -0.800 |
| Arch8 | 0.000 | 0.200 | 0.400 | 0.400 | 0.000 | 0.000 |
| Arch9 | -0.632 | -0.316 | -0.316 | -0.316 | -0.632 | -0.632 |
| Arch10 | 0.400 | -0.400 | -0.200 | -0.200 | 0.400 | 0.400 |
| Arch11 | 0.000 | -0.200 | -0.400 | -0.400 | 0.000 | 0.000 |
| Arch12 | 0.949 | 0.211 | 0.738 | 0.738 | 0.949 | 0.949 |
| Arch13 | **1.000** | 0.400 | 0.800 | 0.800 | **1.000** | **1.000** |
| Arch14 | -0.258 | 0.258 | -0.258 | -0.258 | -0.258 | -0.258 |
| Arch15 | -0.632 | -0.316 | -0.316 | -0.316 | -0.632 | -0.632 |
| Arch16 | -0.400 | -0.400 | -0.200 | -0.200 | -0.400 | -0.400 |
| Arch17 | 0.738 | 0.632 | 0.949 | 0.949 | 0.738 | 0.738 |
| Arch18 | 0.258 | 0.775 | 0.775 | 0.775 | 0.258 | 0.258 |
| Arch19 | 0.775 | -0.258 | 0.258 | 0.258 | 0.775 | 0.775 |
| Arch20 | -0.258 | 0.258 | -0.258 | -0.258 | -0.258 | -0.258 |
| Arch21 | -0.258 | 0.258 | -0.258 | -0.258 | -0.258 | -0.258 |
| Arch22 | 0.258 | 0.775 | 0.775 | 0.775 | 0.258 | 0.258 |
| **AOB** |  |  |  |  |  |  |
| Bact1 | 0.400 | 0.400 | 0.200 | 0.200 | 0.400 | 0.400 |
| Bact2 | -0.400 | 0.400 | 0.200 | 0.200 | -0.400 | -0.400 |
| Bact3 | -0.400 | -0.400 | -0.200 | -0.200 | -0.400 | -0.400 |
| Bact4 | 0.200 | 0.800 | 0.400 | 0.400 | 0.200 | 0.200 |
| Bact5 | **-1.000** | -0.400 | -0.800 | -0.800 | **-1.000** | **-1.000** |
| Bact6 | -0.316 | -0.949 | -0.632 | -0.632 | -0.316 | -0.316 |
| Bact7 | 0.400 | -0.600 | 0.000 | 0.000 | 0.400 | 0.400 |
| Bact8 | 0.800 | 0.800 | **1.000** | **1.000** | 0.800 | 0.800 |
| Bact9 | 0.800 | 0.000 | 0.600 | 0.600 | 0.800 | 0.800 |
| Bact10 | **1.000** | 0.400 | 0.800 | 0.800 | **1.000** | **1.000** |
| Bact11 | **1.000** | 0.400 | 0.800 | 0.800 | **1.000** | **1.000** |
| Bact12 | -0.400 | **-1.000** | -0.800 | -0.800 | -0.400 | -0.400 |
| Bact13 | -0.200 | 0.800 | 0.400 | 0.400 | -0.200 | -0.200 |
| Bact14 | 0.400 | **1.000** | 0.800 | 0.800 | 0.400 | 0.400 |
| Bact15 | 0.200 | 0.800 | 0.400 | 0.400 | 0.200 | 0.200 |
| Bact16 | -0.949 | -0.632 | -0.949 | -0.949 | -0.949 | -0.949 |
| Bact17 | 0.105 | 0.949 | 0.632 | 0.632 | 0.105 | 0.105 |
| Bact18 | -0.258 | 0.258 | -0.258 | -0.258 | -0.258 | -0.258 |
| Bact19 | 0.400 | **1.000** | 0.800 | 0.800 | 0.400 | 0.400 |
| Bact20 | 0.775 | -0.258 | 0.258 | 0.258 | 0.775 | 0.775 |
| Bact21 | 0.775 | -0.258 | 0.258 | 0.258 | 0.775 | 0.775 |
| Bact22 | 0.949 | 0.211 | 0.738 | 0.738 | 0.949 | 0.949 |
| Bact23 | 0.258 | 0.775 | 0.775 | 0.775 | 0.258 | 0.258 |
| Bact24 | 0.258 | 0.775 | 0.775 | 0.775 | 0.258 | 0.258 |
| Bact25 | -0.949 | -0.632 | -0.949 | -0.949 | -0.949 | -0.949 |
| Bact26 | -0.775 | -0.775 | -0.775 | -0.775 | -0.775 | -0.775 |
| Bact27 | -0.211 | 0.316 | 0.316 | 0.316 | -0.211 | -0.211 |
| Bact28 | 0.258 | 0.775 | 0.775 | 0.775 | 0.258 | 0.258 |
| Bact29 | 0.258 | 0.775 | 0.775 | 0.775 | 0.258 | 0.258 |
| Bact30 | 0.775 | -0.258 | 0.258 | 0.258 | 0.775 | 0.775 |
| Bold numbers = *p* values <0.05; underlined numbers = *p* values <0.10 | | | | | | |
