# Supplementary figures and images for: Increases in the soil ammonia oxidizing phylotypes and their rechange due to long-term irrigation with wastewater
Source: PLoS One. 2024 Apr 11;19(4):e0299518. doi: 10.1371/journal.pone.0299518 (PMC11008854; doi:10.1371/journal.pone.0299518)

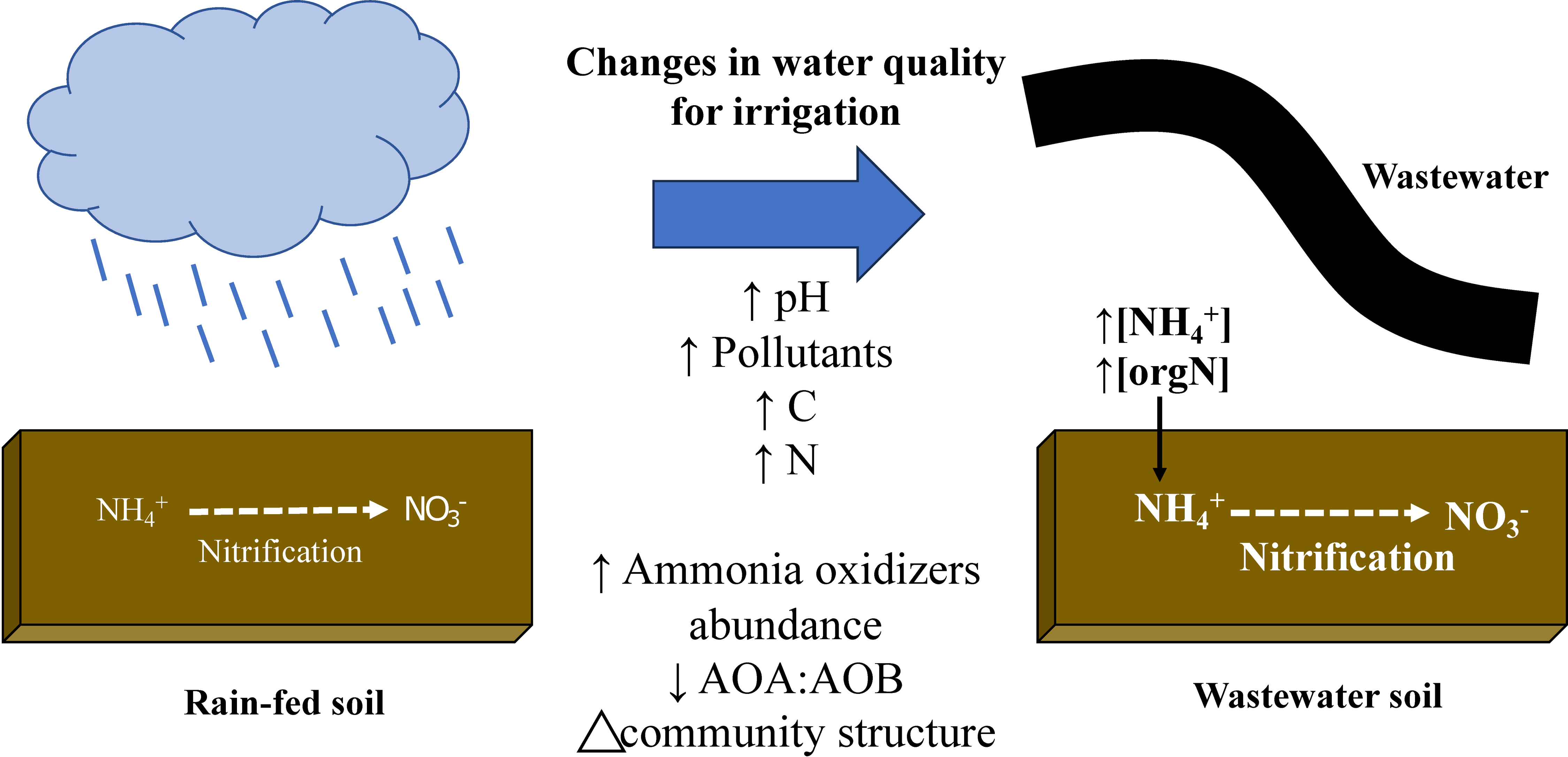

Supplement: S1 Graphical abstract — (TIF) [file pone.0299518.s003.tif]
